# Supplementary material for: A Global Screen for Assembly State Changes of the Mitotic Proteome by SEC-SWATH-MS
Source: Cell Syst. 2020 Feb 26;10(2):133–155.e6. doi: 10.1016/j.cels.2020.01.001 (PMC7042714; doi:10.1016/j.cels.2020.01.001)

Q99714 | HCD2_HUMAN | HSD17B10 ERAB HADH2 MRPP2 SCHAD SDR5C1 XH98G2
Monomer MW [kDa]: 26.923 Monomer expected elution fraction: 52

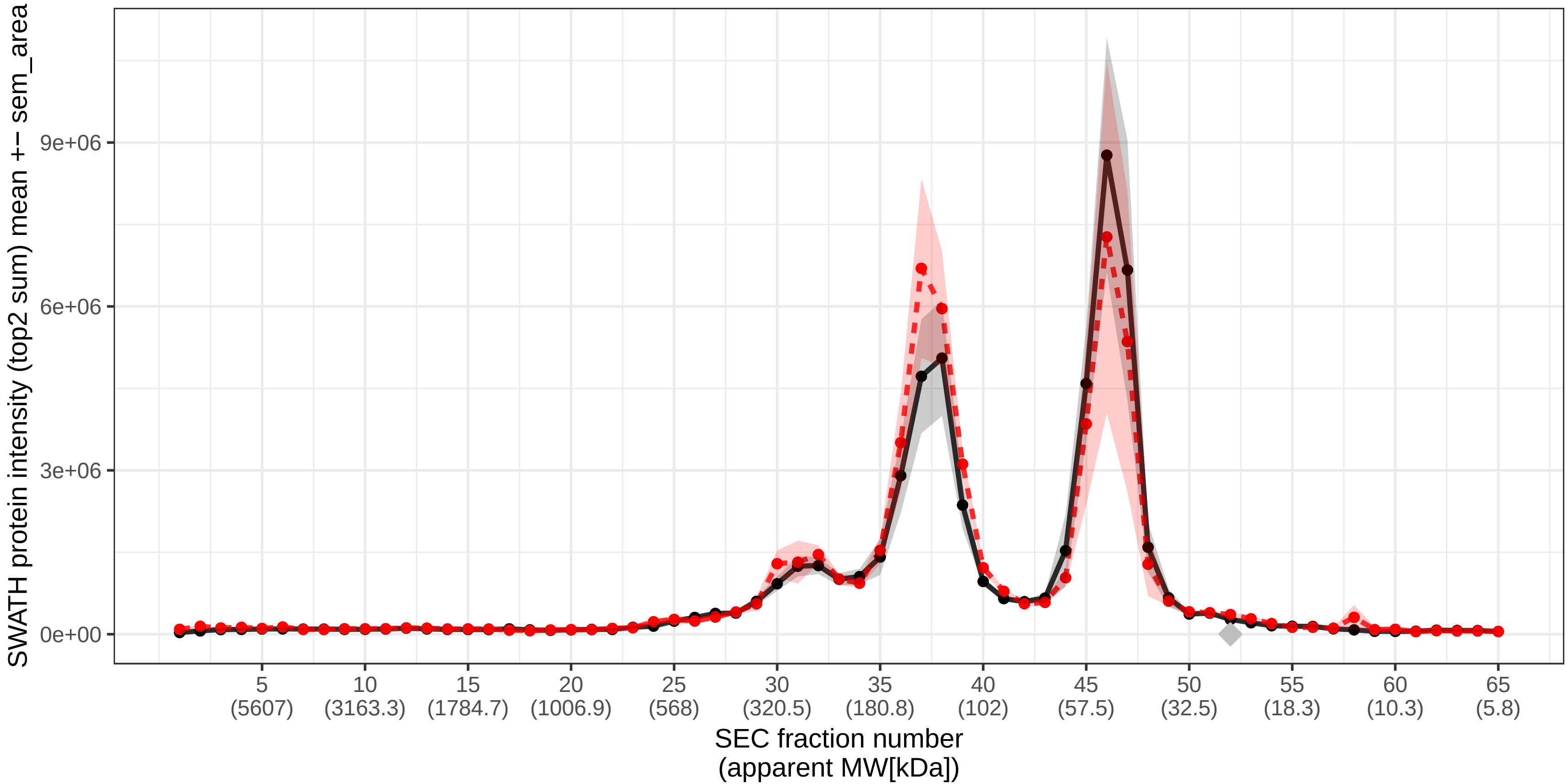

Supplement: Data S1. SEC-SWATH-MS Protein Chromatograms, Related to Figure 1 [file mmc6.zip › SECchrom_Q99714_HCD2_HUMAN_HSD17B10_ERAB_HADH2_MRPP2_SCHAD_.pdf]
